# Supplementary material for: Cost-benefit analysis of a trifocal intraocular lens versus a monofocal intraocular lens from the patient’s perspective in the United States
Source: PLoS One. 2022 Nov 3;17(11):e0277093. doi: 10.1371/journal.pone.0277093 (PMC9632823; doi:10.1371/journal.pone.0277093)
Supplement: S1 File — (PDF) [file pone.0277093.s001.pdf]

S1 File. Detailed calculations of the cost-benefit study.

1. **Cost inputs**
   a. Cost of Monofocal (SN60AT) bilateral IOL procedure was calculated as follows:
      i. Physician fee ($557.58) + ASC fee ($1,012.72) + 1 office visit ($152.66) = $1722.96 x 15% patient co-insurance = $258.44/eye x 2 eyes = $516.89
   b. Cost of IOL explantation was calculated as follows:
      i. Physician fee ($925.34) + ASC fee ($1,012.72) + 1 office visit ($128.12) = $2,066.18 x 15% patient co-insurance = $309.93/eye
   c. Cost of YAG laser capsulotomy was calculated as follows:
      i. Physician fee ($317.95) + 1 office visit ($128.12) = $446.07 x 15% patient co-insurance = $66.91
   d. Cost of glasses per year
      i. Total glasses costs in TFNT00 IOL: Calculated by multiplying the proportion of patients with reading/distance/bifocal/progressive glasses in each cycle* cost of respective spectacle type = $81
      ii. Total glasses costs in SN60AT IOL: Calculated by multiplying the proportion of patients with reading/distance/bifocal/progressive glasses in each cycle* cost of respective spectacle type = $2,674
2. **Base-case results**
   a. Total lifetime costs incurred by patients implanted with TFNT00 was calculated as follows:
      i. IOL Procedure Cost: Clinician input ($3,000/eye) = $6,000

ii. IOL Explantation Cost: Calculated by multiplying the TFNT00 IOL explantation (0.008) * Cost of IOL explantation ($309.93) = $2

iii. Eye Drops Cost: Eye drops costs were applied to all patients who underwent TFNT00 IOL implantation plus patients with TFNT00 IOL explantation i.e., (1+0.008) * $23 = $23

iv. YAG laser capsulotomy cost: Calculated by multiplying the transition probabilities of YAG capsulotomy (0.248) * Cost of YAG laser capsulotomy ($66.91) = $17

v. Optometrists Visit Costs: Calculated by multiplying the proportion of patients with glasses in each cycle * cost of optometrist visit = $14

vi. Factoring in the costs of each input in Table 3 with the transition probabilities (Table 1) and distribution of spectacle need (Table 2) for TFNT00 in the model, the total lifetime costs equal $6,137.

b. Total lifetime costs incurred by patients implanted with SN60AT was calculated as follows:

i. IOL Procedure Cost: Cost of SN60AT ($258.44/eye) = $517

ii. IOL Explantation Cost: Calculated by multiplying the SN60AT IOL explantation (0.009) * Cost of IOL explantation ($309.93) = $3

iii. Eye Drops Cost: Eye drops costs were applied to all patients who underwent SN60AT IOL implantation plus patients with SN60AT IOL explantation i.e., (1+0.009) * $23 = $23

iv. YAG laser capsulotomy cost: Calculated by multiplying the transition probabilities of YAG capsulotomy (0.061) * Cost of YAG laser capsulotomy ($66.91) = $4

v. Optometrists Visit Costs: Calculated by multiplying the proportion of patients with glasses in each cycle * cost of optometrist visit = $133

vi. Factoring in the costs of each input in Table 3 with the transition probabilities (Table 1) and distribution of spectacle need (Table 2) for SN60AT in the model, the total lifetime costs equal $3,354.

c. The average lifetime spectacles cost savings with bilateral TFNT00 IOL compared to those implanted with SN60AT IOL was calculated as follows:

Total Glasses Costs: TFNT00 IOL ($81) – SN60AT IOL ($2,674): -$2,593

d. Total quality adjusted life-year (QALYs) per patient for each arm was calculated as follows:

i. Well health state: LYs spent in well health state * utility of well state

ii. Glasses health state: LYs spent in glasses only health state * (utility of well health state + dis-utility of glasses health sate)

iii. Glare/haloes/starburst health state: LYs spent in glare/haloes/starburst health state * (utility of well health state + dis-utility of glares*(duration of Glare/Haloes/Starbursts (in days) (60.9 days) /365.25))

iv. Glare/haloes/starburst health with glasses: LYs spent in glare/haloes/starburst health with glasses * (utility of well health state + dis-

utility of glasses + dis-utility of glare*(duration of Glare/Haloes/Starbursts (in days) (60.9 days) /365.25))

v. IOL explantation: Frequency of IOL explantation * dis-utility of IOL exchange

The sum of these results equals 13.09 QALYs in TFNT00 IOL arm.

The sum of these results equals 12.41 QALYs in SN60AT IOL arm.

e. At a willingness-to-pay (WTP) threshold of $50,000 per QALY gain, lifetime net monetary benefit (NMB) per patient with bilateral TFNT00 IOL implantation was calculated as:

   i. WTP ($50,000) x incremental QALY gain (0.67) – total incremental costs ($2,783) = $30,941

3. Sensitivity analysis

a. Results from the probabilistic sensitivity analysis (PSA) confirmed the robustness of the base-case deterministic results as the average NMB from 1,000 simulations was estimated to be $30,875, which was similar to the deterministic base-case NMB. For model parameters bounded by 0 and 1, the beta distribution was used to vary the parameter using the reported mean and the number of observations. For cost and other parameters whose values are greater than 0, the log-normal distribution was used to vary the parameter based on the mean and standard error of the parameter. If standard error of any parameters is not available, it was assumed equal to 20% of mean value of the parameter. Normal distribution was used to vary disutilities. Dirichlet distribution was used for multivariate probability parameters. Details are provided in the Table below:

Parameters	Deterministic value	Distribution	N/SE	Source
Time Horizon (in years)	30	NA	NA	
Discount rate for costs	3%	NA	NA	
Discount rate for benefits	3%	NA	NA	
Multifocal: Overall spectacle dependence with multifocal IOL	19.5%	Beta	129	Modi et al [10]
Multifocal: Glare and/or haloes and/or starbursts with multifocal IOL	12.0%	Beta	129	AcrySof IQ PanOptix Directions for Use [15]
Multifocal: Resolution of glare and/or haloes	81.0%	Beta	129	Hu et al [11]
Multifocal: IOL explantation	0.8%	Beta	129	Modi et al [10]
Multifocal: Average YAG use per patient	24.8%	Beta	129	Data on file [13]
Monofocal: Overall spectacle dependence with monofocal IOL	92.0%	Beta	114	Modi et al [10]
Monofocal: Glare and/or haloes and/or starbursts with monofocal IOL	7.2%	Beta	114	AcrySof IQ PanOptix Directions For Use [15]
Monofocal: Resolution of glare and/or haloes	81.0%	Beta	114	Hu et al [11]
Monofocal: IOL explantation	0.9%	Beta	114	Modi et al [10]
Monofocal: Average YAG use per patient	6.1%	Beta	114	Data on file [13]
Multifocal: Reading glasses	75.0%	Dirichlet	129	Data on file [13] and clinical input
Multifocal: Distance glasses	20.0%	Dirichlet	129	Data on file [13] and clinical input
Multifocal: Bi-focal glasses	2.5%	Dirichlet	129	Data on file [13] and clinical input
Multifocal: Progressive glasses	2.5%	Dirichlet	129	Data on file [13] and clinical input
Monofocal: Reading glasses	40.0%	Dirichlet	114	Data on file [13] and clinical input
Monofocal: Distance glasses	10.0%	Dirichlet	114	Data on file [13] and clinical input
Monofocal: Bi-focal glasses	25.0%	Dirichlet	114	Data on file [13] and clinical input
Monofocal: Progressive glasses	25.0%	Dirichlet	114	Data on file [13] and clinical input
Multifocal: Proportion of Reading glasses patients receiving Lasik surgery for vision correction	50.0%	Beta	129	Clinical input
Multifocal: Proportion of Distance glasses patients receiving Lasik surgery for vision correction	75.0%	Beta	129	Clinical input

Parameters	Deterministic value	Distribution	N/SE	Source
Multifocal: Proportion of Bi-focal glasses patients receiving Lasik surgery for vision correction	75.0%	Beta	129	Clinical input
Multifocal: Proportion of Progressive glasses patients receiving Lasik surgery for vision correction	75.0%	Beta	129	Clinical input
Monofocal: Proportion of Reading glasses patients receiving Lasik surgery for vision correction	0.0%	Beta	114	Clinical input
Monofocal: Proportion of Distance glasses patients receiving Lasik surgery for vision correction	0.0%	Beta	114	Clinical input
Monofocal: Proportion of Bi-focal glasses patients receiving Lasik surgery for vision correction	0.0%	Beta	114	Clinical input
Monofocal: Proportion of Progressive glasses patients receiving Lasik surgery for vision correction	0.0%	Beta	114	Clinical input
Efficacy of Lasik surgery for Vision Correction	90.0%	Beta	243	Clinical input
Well	1.00	Beta	243	Hu et al [11]
Utility decrement: Glare and/or haloes and/or starbursts	-0.18	Normal	SE as % of mean: 20%	Brown et al [25]
Utility decrement: Wearing glass	-0.07	Normal	SE as % of mean: 20%	Dobrez and Calhoun [26]
Utility decrement: IOL explantation	-0.15	Normal	SE as % of mean: 20%	Busbee et al [4]
Duration of Glare/Haloes/Starbursts (in days)	60.88	Log-normal	12.2	Jin et al [27] and clinical input
Cost of Monofocal IOL procedure per eye	$258	Log-normal	SE as % of mean: 20%	CPT codes 66984, 92004
Cost of Multifocal IOL procedure per eye	$3,000	Log-normal	SE as % of mean: 20%	All About Vision [19] and clinical input
Cost of IOL explantation	$310	Log-normal	SE as % of mean: 20%	CPT codes 66986, 92014
Cost of Eye Drops	$23	Log-normal	SE as % of mean: 20%	Drugs.com [23]
Optometrist visit	$11	Log-normal	SE as % of mean: 20%	CPT code 99213
Cost of YAG laser capsulotomy	$67	Log-normal	SE as % of mean: 20%	CPT codes 66821, 92014
Frequency of visit to Optometrist for eye check-up	1.00	NA	SE as % of mean: 20%	NVISION [22]
Cost of Reading glasses	$15	Log-normal	SE as % of mean: 20%	Walmart.com [21]

Parameters	Deterministic value	Distribution	N/SE	Source
Cost of Distance glasses	$126	Log-normal	SE as % of mean: 20%	Walmart.com [21]
Cost of Bi-focal glasses	$500	Log-normal	SE as % of mean: 20%	Market research data [20]
Cost of Progressive glasses	$500	Log-normal	SE as % of mean: 20%	Market research data [20]
Time for glasses replacement (in years): Reading Glasses	1.25	Log-normal	SE as % of mean: 20%	NVISION [22] and clinical input
Time for glasses replacement (in years): Distance Glasses	1.25	Log-normal	SE as % of mean: 20%	NVISION [22] and clinical input
Time for glasses replacement (in years): Bi-focal Glasses	1.25	Log-normal	SE as % of mean: 20%	NVISION [22] and clinical input
Time for glasses replacement (in years): Progressive Glasses	1.25	Log-normal	SE as % of mean: 20%	NVISION [22] and clinical input

i. A key output of a PSA is the proportion of results that fall favorably (i.e. considered cost effective) in relation to a given cost-effectiveness threshold, which is represented in the cost effectiveness curve. The cost-effectiveness acceptability curve presented in Figure 3 projected that at a WTP threshold of $5,000, $10,000 and $12,000 TFNT00 IOL would be cost effective 71%, 99% and 100% respectively.

4. **Scenario analyses**

   a. Scenario analyses were conducted by varying single model parameter/setting of interest at a time and keeping all other parameters/settings unchanged. The impact of relevant parameter/setting on model results (ICER) were recorded for each scenario and then plotted on a graph which is presented in figure 4 of the manuscript.
