# Supplementary material for: Cost-benefit analysis of a trifocal intraocular lens versus a monofocal intraocular lens from the patient’s perspective in the United States
Source: PLoS One. 2022 Nov 3;17(11):e0277093. doi: 10.1371/journal.pone.0277093 (PMC9632823; doi:10.1371/journal.pone.0277093)
Supplement: Attachment — Submitted filename: Response to Reviewers_PanOptix US CBA Manuscript_PLOS ONE.docx [file pone.0277093.s002.docx]

Editors

PLOS ONE

September 15, 2022

Dear Editors,

We thank you and the reviewers for considering our manuscript entitled “Cost-benefit analysis of a trifocal intraocular lens versus a monofocal intraocular lens from the patient's perspective in the United States” (ID PONE-D-22-16771) and providing valuable comments to further improve this manuscript.

We have made efforts to address reviewer comments and revised the manuscript accordingly. Please refer to the table below summarizing author response to reviewer comments. We also attached the revised manuscript in track change and clean version formats for your perusal.

Please address all correspondence concerning this manuscript to me at johnberdahl@gmail.com.

Thank you for considering our work.

Sincerely,

Dr. John Berdahl

Table 1: Reviewer Comments and Authors’ Response

| **#** | **Comment** | **Author Response** |
| --- | --- | --- |
| Editor comment #1 | Please ensure that your manuscript meets PLOS ONE's style requirements, including those for file naming. | Thank you for your comment. As requested, the title page and full manuscript have been updated per PLOS ONE’s style requirements. |
| Editor comment #2 | Thank you for stating the following financial disclosure:  " This study was sponsored by Alcon Vision LLC, Fort Worth, Texas, USA ([https://www.alcon.com/](https://nam12.safelinks.protection.outlook.com/?url=https%3A%2F%2Fwww.alcon.com%2F&data=05%7C01%7CKayla.Mills%40alcon.com%7C6aa012354e624b65273208da760fbac7%7C34cd94b5d86c447f8d9b81b4ff94d329%7C0%7C0%7C637952107333247156%7CUnknown%7CTWFpbGZsb3d8eyJWIjoiMC4wLjAwMDAiLCJQIjoiV2luMzIiLCJBTiI6Ik1haWwiLCJXVCI6Mn0%3D%7C3000%7C%7C%7C&sdata=v90rhPhwB2ggZxmbahs1SzGtTMznwRqYLvL7TBh38w0%3D&reserved=0)). JB and CB received consulting fees from Alcon. MD is an employee of Alcon Vision LLC (the study sponsor). HR and RG are employees of Skyward Analytics Pte. Ltd. and Skyward Analytics Pvt. Ltd., respectively, and received consulting fee from Alcon to conduct this study. The authors report no other conflicts of interest related to this work."  At this time, please address the following queries:  a)        Please clarify the sources of funding (financial or material support) for your study. List the grants or organizations that supported your study, including funding received from your institution.  b)        State what role the funders took in the study. If the funders had no role in your study, please state: “The funders had no role in study design, data collection and analysis, decision to publish, or preparation of the manuscript.”  c)        If any authors received a salary from any of your funders, please state which authors and which funders.  d)        If you did not receive any funding for this study, please state: “The authors received no specific funding for this work.”  Please include your amended statements within your cover letter; we will change the online submission form on your behalf. | Thank you for your comments. Based on the feedback provided, we have updated the financial disclosure as follows:  This study was funded by Alcon Vision LLC, Fort Worth, Texas, USA, the manufacturer of AcrySof IQ PanOptix and AcrySof SN60AT IOLs. JB and CB received consulting fees from Alcon. MD is a full-time employee of Alcon Vision LLC (the study sponsor). HR and RG are employees of Skyward Analytics Pte. Ltd. and Skyward Analytics Pvt. Ltd., respectively, and received consulting fees from Alcon to conduct this study. All authors supported study design, data collection, analysis, and preparation of the manuscript. No authors received remuneration for writing this manuscript. The authors report no other conflicts of interest related to this work. |
| Editor comment #3 | In your Data Availability statement, you have not specified where the minimal data set underlying the results described in your manuscript can be found. PLOS defines a study's minimal data set as the underlying data used to reach the conclusions drawn in the manuscript and any additional data required to replicate the reported study findings in their entirety. All PLOS journals require that the minimal data set be made fully available. For more information about our data policy, please see [http://journals.plos.org/plosone/s/data-availability](https://nam12.safelinks.protection.outlook.com/?url=http%3A%2F%2Fjournals.plos.org%2Fplosone%2Fs%2Fdata-availability&data=05%7C01%7CKayla.Mills%40alcon.com%7C6aa012354e624b65273208da760fbac7%7C34cd94b5d86c447f8d9b81b4ff94d329%7C0%7C0%7C637952107333247156%7CUnknown%7CTWFpbGZsb3d8eyJWIjoiMC4wLjAwMDAiLCJQIjoiV2luMzIiLCJBTiI6Ik1haWwiLCJXVCI6Mn0%3D%7C3000%7C%7C%7C&sdata=PZIu2ZzWh15HXfu5%2BnrQnlaxw6TqYVV9myBW7EUTjlI%3D&reserved=0).  Upon re-submitting your revised manuscript, please upload your study’s minimal underlying data set as either Supporting Information files or to a stable, public repository and include the relevant URLs, DOIs, or accession numbers within your revised cover letter. For a list of acceptable repositories, please see [http://journals.plos.org/plosone/s/data-availability#loc-recommended-repositories](https://nam12.safelinks.protection.outlook.com/?url=http%3A%2F%2Fjournals.plos.org%2Fplosone%2Fs%2Fdata-availability%23loc-recommended-repositories&data=05%7C01%7CKayla.Mills%40alcon.com%7C6aa012354e624b65273208da760fbac7%7C34cd94b5d86c447f8d9b81b4ff94d329%7C0%7C0%7C637952107333247156%7CUnknown%7CTWFpbGZsb3d8eyJWIjoiMC4wLjAwMDAiLCJQIjoiV2luMzIiLCJBTiI6Ik1haWwiLCJXVCI6Mn0%3D%7C3000%7C%7C%7C&sdata=FIR3GQY0vm%2Bkf%2F6T2Uhq9N8bN%2FfCKIjGh03uC482HWM%3D&reserved=0). Any potentially identifying patient information must be fully anonymized.  Important: If there are ethical or legal restrictions to sharing your data publicly, please explain these restrictions in detail. Please see our guidelines for more information on what we consider unacceptable restrictions to publicly sharing data: [http://journals.plos.org/plosone/s/data-availability#loc-unacceptable-data-access-restrictions](https://nam12.safelinks.protection.outlook.com/?url=http%3A%2F%2Fjournals.plos.org%2Fplosone%2Fs%2Fdata-availability%23loc-unacceptable-data-access-restrictions&data=05%7C01%7CKayla.Mills%40alcon.com%7C6aa012354e624b65273208da760fbac7%7C34cd94b5d86c447f8d9b81b4ff94d329%7C0%7C0%7C637952107333247156%7CUnknown%7CTWFpbGZsb3d8eyJWIjoiMC4wLjAwMDAiLCJQIjoiV2luMzIiLCJBTiI6Ik1haWwiLCJXVCI6Mn0%3D%7C3000%7C%7C%7C&sdata=zyLt6Nv2jZyAZCtL%2F3CnRlcjeVPR4TJfa873eSaEdd8%3D&reserved=0). Note that it is not acceptable for the authors to be the sole named individuals responsible for ensuring data access.  We will update your Data Availability statement to reflect the information you provide in your cover letter. | Thank you for your comment. The study’s minimal data set has been uploaded with this resubmission. |
| Editor comment #4 | PLOS requires an ORCID iD for the corresponding author in Editorial Manager on papers submitted after December 6th, 2016. Please ensure that you have an ORCID iD and that it is validated in Editorial Manager. To do this, go to ‘Update my Information’ (in the upper left-hand corner of the main menu), and click on the Fetch/Validate link next to the ORCID field. This will take you to the ORCID site and allow you to create a new iD or authenticate a pre-existing iD in Editorial Manager. | Thank you for your comment. I have followed the appropriate steps in which I received the ORCID iD and validated it in Editorial Manager.  ORCID iD: 0000-0002-5468-7405 |
| Editor comment #5 | While revising your submission, please upload your figure files to the Preflight Analysis and Conversion Engine (PACE) digital diagnostic tool, [https://pacev2.apexcovantage.com/](https://nam12.safelinks.protection.outlook.com/?url=https%3A%2F%2Fpacev2.apexcovantage.com%2F&data=05%7C01%7CKayla.Mills%40alcon.com%7C6aa012354e624b65273208da760fbac7%7C34cd94b5d86c447f8d9b81b4ff94d329%7C0%7C0%7C637952107333247156%7CUnknown%7CTWFpbGZsb3d8eyJWIjoiMC4wLjAwMDAiLCJQIjoiV2luMzIiLCJBTiI6Ik1haWwiLCJXVCI6Mn0%3D%7C3000%7C%7C%7C&sdata=38M5y%2FkRIvOxZ15hSci7ihIONF1DWbAvlPx2BDYu2Jw%3D&reserved=0). PACE helps ensure that figures meet PLOS requirements. To use PACE, you must first register as a user. Registration is free. Then, login and navigate to the UPLOAD tab, where you will find detailed instructions on how to use the tool. If you encounter any issues or have any questions when using PACE, please email PLOS at [figures@plos.org](mailto:figures@plos.org). Please note that Supporting Information files do not need this step. | Thank you for your comment. The figures have now been uploaded to the PACE tool and adjusted accordingly. |
| Reviewer 1 comment #1 | The manuscript writing is in general well-written and easy to follow. However, a severe limitation of the paper is reliance on assumption/clinical input: the majority of input parameters for the study are assumed. For instance, the entirety of Table 2 is based on “clinical input.” I recommend the authors conduct literature searches and/or primary data analyses to generate reasonable parameter estimates in lieu of powering the model almost entirely based on assumption/clinical opinion. | Thank you for your comment and recommendation. Based on this feedback, we have thoroughly reviewed published literature to support our assumptions and have made significant adjustments throughout the manuscript. For example, Table 2 now reflects data that was derived from the Modi et al study and verified by clinical expertise, then normalized to equal 100%. Furthermore, for parameters which were not supported by literature, we have acknowledged this as a limitation of the analysis and recommended this as an area for future research (lines 379-385, pages 20-21 of the tracked change version):  “Moreover, surgeon real-world clinical experience was used to fill gaps in input parameters to estimate the rate and effectiveness of LASIK enhancement as well as the development and resolution rates of visual disturbances. Authors recommend generating clinical evidence on these parameters in future studies.” |
| Reviewer 1 comment #2 | The article often refers to the analysis as a “cost-benefit” analysis, but the way the study is conducted, “cost-effectiveness” analysis is more appropriate. | Thank you for your comment. We agree with reviewer comments that the methodology used in our study appears broadly similar to a cost-effectiveness or cost-utility study, however one key difference which justifies labelling our approach as a cost-benefit analysis is due to the fact that we have estimated both benefits and costs in monetary units [supported by ref 1,2]. Moreover, this approach is suitable to represent the US healthcare system scenario where patients’ pay out of pocket to receive presbyopia (and astigmatism) correcting intraocular lenses [3], while it is not covered by the US Centers for Medicare & Medicaid Services (CMS) [4,5].  [1] Sanders GD, Neumann PJ, Basu A, Brock DW, Feeny D, Krahn M, Kuntz KM, Meltzer DO, Owens DK, Prosser LA, Salomon JA. Recommendations for conduct, methodological practices, and reporting of cost-effectiveness analyses: second panel on cost-effectiveness in health and medicine. Jama. 2016 Sep 13;316(10):1093-103.  [2] Turner HC, Archer RA, Downey LE, Isaranuwatchai W, Chalkidou K, Jit M, Teerawattananon Y. An introduction to the main types of economic evaluations used for informing priority setting and resource allocation in healthcare: key features, uses, and limitations. Frontiers in Public Health. 2021:1236.  [3] Kim DD, Silver MC, Kunst N, Cohen JT, Ollendorf DA, Neumann PJ. Perspective and costing in cost-effectiveness analysis, 1974–2018. Pharmacoeconomics. 2020 Oct;38(10):1135-45.  [4] Department of Health and Human Services. CMS Rulings: Ruling No. 05-01. www.cms.gov/Regulations-and-Guidance/Guidance/Rulings/downloads/cmsr0501.pdfMay 3, 2005. Accessed 6 Sept 2022.  [5] Department of Health and Human Services. CMS Rulings: Ruling No. CMS-1536-R. www.cms.gov/Regulations-and-Guidance/Guidance/Rulings/downloads/CMS1536R.pdfJanuary 22, 2007. Accessed 6 Sept 2022. |
| Reviewer 1 comment #3 | Cost-effectiveness analyses from the patient perspective are uncommon. How is the threshold of $50,000 willingness to pay per quality-adjusted life year (QALY) informed? | Thank you for your comment. We acknowledge the reviewer’s point, however as stated above, it is the right approach given US patients have to pay out of pocket to receive presbyopia and/or astigmatism correcting IOLs such as AcrySof IQ PanOptix IOL. We have presented both cost and outcomes in monetary terms as is the key requirement for a cost-benefit analysis. Our reasoning for using the $50,000/QALY WTP threshold is supported by ICER’s Value Assessment Framework [1] with a suggested range of $50,000-$150,000/QALY gain, as well as from an analysis on QALY by Braithwaite et al, which explains that patients are willing to pay $50,000 and even up to $200,000 per QALY gain [2]. Therefore, a more stringent threshold of $50,000/QALY was used, while the findings were also extrapolated to higher WTP thresholds. We have further clarified this in the discussion section of the manuscript (lines 328-332, page 18):  “ICER suggests a range of $50,000-$150,000/QALY gain for the WTP threshold for the economic evaluation of novel treatments [28]. The WTP threshold of $50,000/QALY was used in this study to reflect the patient perspective of the health care system, bearing in mind that patients nowadays are willing to pay up to $100,000-$200,000/QALY.”  [1] ICER 2020-2023 Value Assessment Framework. Accessed May 10, 2021. <https://icer.org/wp-content/uploads/2021/03/ICER_2020_2023_VAF_013120-4-2.pdf>.  [2] Braithwaite RS, Meltzer DO, King Jr JT, Leslie D, Roberts MS. What does the value of modern medicine say about the $50,000 per quality-adjusted life-year decision rule?. Medical care. 2008 Apr 1:349-56. |
| Reviewer 1 comment #4 | Have the authors conducted a literature review of previous economic evaluations in this disease area? A paragraph in the introduction section should discuss this. | Thank you for your comment and suggestion. There are a couple of cost-effectiveness analyses that have been performed to explain the benefits of AT-IOLs in general in comparison to monofocal IOLs. We agree with the reviewer that it would be important to highlight previous work done, thus we have included this in the introduction section (lines 83-86, page 4):  “Some studies have reported economic evaluations of AT-IOL versus monofocal IOL options [3,11-12]; however, there currently is no published evidence on the cost benefits of TFNT00 IOL in the US.” |
| Reviewer 1 comment #5 | Model inputs – the transition probabilities in Table 1 do not appear to be converted to fit the Markov model (i.e., converted to yearly cycles). | Thank you for your comment. The transition probabilities in Table 1 are applicable for Year 1 only and applied one time in the first cycle of the model, except for resolution of visual disturbances which is applied in Year 2 (second cycle). Thereafter, patients transition to death or remain in their respective health state as per US life tables. Based on the previously published study Hu et al 2019 and per clinical expertise, we assumed that chances of developing bothersome visual disturbances and the need for spectacles would occur within the first-cycle post-cataract surgery. Therefore, the observed proportion of spectacle dependence and severe visual disturbances in the clinical trial were used as an annual transition probability in the economic model. We have made the relevant edits in Table 1 of the manuscript (line 141, pages 6-7) and in the text (lines 118-122, pages 5-6):  “Transition probabilities were used to map out the different health states patients may experience post-cataract surgery, and are applied one time in the first yearly cycle of the model, except for resolution of visual disturbances which is applied in the second cycle, following the first year (see Table 1).” |
| Reviewer 1 comment #6 | Disutilities – a utility value of 1 (equivalent to perfect health) in the well state is not clinically reasonable given the advanced age and comorbidity status of the patient population of interest. | Thank you for your comment. The utility value of 1 was intended to represent perfect vision-related health, i.e. patients that do not require glasses and do not experience any visual disturbances. This also reflects the utility value used in the Hu et al study. After reviewing, we updated lines 194-198 (page 12) to provide further clarity of why we chose the utility value of 1 for the ‘well’ health state:  “In this model, the “well” health state represents patients with optimal vision-related health (complete spectacle independence at all distances as proxy for optimized near, intermediate, and far distance visual acuity and absence of any visual disturbances). Therefore, the vision-related utility of the “well” health state was assumed to be 1, which reflects a similar approach to the Hu et al study [11].” |
| Reviewer 1 comment #7 | Probabilistic sensitivity analyses – how were distributions and parameters chosen for each input? What are these distributions? | Thank you for your comments and questions. We have added text to the manuscript to provide clarity on this (lines 243-249, page 14):  “For model parameters bounded by 0 and 1, the beta distribution was used to vary the parameter using the reported mean and the number of observations. For cost and other parameters whose values are greater than 0, the log-normal distribution was used to vary the parameter based on the mean and standard error of the parameter. If standard error was not available, it was assumed to equal 20% of the mean value of the parameter. Normal distribution was used to vary disutilities. Dirichlet distribution was used for multivariate probability parameters.” |
| Reviewer 1 comment #8 | Scenario analyses – how do the authors reconcile scenario analyses starting at ages 50, 55, and 60 if many inputs are Medicare-specific? | Thank you for your comment. We agree with the author that ages 50, 55 and 60 are not as relevant as our analysis focuses on the Medicare population (generally 65 years and older). Therefore, we updated the scenario analysis to include only age 65 to align with our parameters. This has been updated in Figure 4 and in the scenario analysis section of the manuscript (lines 251-256, page 14):  “The following model settings were varied in the model: time horizon (5, 10, 15, 20, and 25 years), patients’ age (65 years old), and discount rates (0% and 5%).” |
| Reviewer 1 comment #9 | Discussion – the Institute for Clinical and Economic Review is an independent organization. It is not appropriate to follow their guidelines for economic evaluation in academic work. I would recommend the authors follow recommendations of the Second Panel in Cost-Effectiveness in Health and Medicine instead. | Thank you for your comment and recommendation. The framework we used from the US Institute for Clinical and Economic Review (ICER) includes analyses performed according to the highest academic methodological standards, and reports sources of evidence, strengths and limitations, and further details. ICER has been both endorsed by AMCP and ISPOR, and its guidelines have been through peer-reviewed journals. Therefore, we believe ICER provides a strong framework to follow and clear explanation for our use in both methodological approach and WTP threshold [ICER 2020-2023 Value Assessment Framework. Accessed May 10, 2021. https://icer.org/wp-content/uploads/2021/03/ICER_2020_2023_VAF_013120-4-2.pdf]. |
| Reviewer 2 comment #1 | This study attempts to validate the implantation of the Alcon PanOptix IOL based on QALY methodology. The problem in this is the unknown long-term quality of vision with this lens, knowing that the material is subject to the development of subsurface nanoglistenings and, to a lesser extent, glistenings. Furthermore, long-term refractive stability is uncertain, knowing the ongoing change in corneal astigmatism with aging. | Thank you for your comments. We agree with the reviewer that long-term quality of vision and refractive stability outcomes with these IOLs are currently not reported in the literature. However, we would like to acknowledge that intraocular lenses based on AcrySof IQ biomaterial first became available more than 25 years ago, and PanOptix has been implanted in over 1 million eyes (Alcon Data on File REF-13513, 2021.) To authors’ knowledge, to date IOLs made from AcrySof IQ biomaterial and the PanOptix IOL have not received any regulatory safety/warning letter pertaining to its lack of efficacy or safety, such as those related to quality of vision, sub-surface nano-glistening, glistening, or long-term refractive stability.  Recognizing the reviewer’s comment, since we are limited in research data available that support the long-term efficacy of multifocal IOLs, we believe this could be an interesting study to consider for future research and should be addressed in succeeding economic evaluations. Therefore, we have included this in the discussion section (lines 364-365, page 20):  “Future studies should be considered to evaluate the long-term efficacy of multifocal IOLs.” |
| Reviewer 2 comment #2 | There are a large number of editing errors in the manuscript. | Thank you for your comment. We have thoroughly reviewed the manuscript and have made edits throughout. |
